# Analysis of anesthesia-controlled operating room time after propofol-based total intravenous anesthesia compared with desflurane anesthesia in functional endoscopic sinus surgery

**DOI:** 10.1097/MD.0000000000009805

**Published:** 2018-02-02

**Authors:** Tien-Chien Liu, Hou-Chuan Lai, Chueng-He Lu, Yuan-Shiou Huang, Nan-Kai Hung, Chen-Hwan Cherng, Zhi-Fu Wu

**Affiliations:** a Division of Anesthesiology, Zouying Branch of Kaohsiung Armed Force General Hospital, Kaohsiung; b Department of Anesthesiology, Tri-Service General Hospital and National Defense Medical Center, Taipei, Taiwan, Republic of China.

**Keywords:** anesthesia controlled time, desflurane, functional endoscopic sinus surgery, propofol

## Abstract

Anesthesia technique may contribute to the improvement of operation room (OR) efficiency by reducing anesthesia-controlled time. We compared the difference between propofol-based total intravenous anesthesia (TIVA) and desflurane anesthesia (DES) for functional endoscopic sinus surgery (FESS) undergoing general anesthesia

We performed a retrospective study using data collected in our hospital to compare the anesthesia-controlled time of FESS using either TIVA via target-controlled infusion with propofol/fentanyl or DES/fentanyl-based anesthesia between January 2010 and December 2011. The various time intervals (surgical time, anesthesia time, extubation time, total OR stay time, post anesthesia care unit [PACU] stay time) and the percentage of prolonged extubation were compared between the 2 anesthetic techniques.

We included data from 717 patients, with 305 patients receiving TIVA and 412 patients receiving DES. An emergence time >15 minutes is defined as prolonged extubation. The extubation time was faster (8.8 [3.5] vs. 9.6 [4.0] minutes; *P* = .03), and the percentage of prolonged extubation was lower (7.5% vs. 13.6%, risk difference 6.1%, *P* < .001) in the TIVA group than in the DES group. However, there was no significant difference between ACT, total OR stay time, and PACU stay time.

In our hospital, propofol-based TIVA by target-controlled infusion provide faster emergence and lower chance of prolonged extubation compared with DES anesthesia in FESS. However, the reduction in extubation time may not improve OR efficiency.

## 1 Introduction

Healthcare systems in current days are facing significant problems of increasing expenditures. As a result of the need to increase efficiency with cost containment, the stress on optimizing operation room (OR) efficiency increased. OR efficiency is regulated by several factors; 2 of the most important factors are anesthesia-controlled time (ACT) and turnover time.^[1]^ The time required between the end of surgery till extubation is of special interest to anesthesiologists because it is affected by anesthesia agents administrated.^[2–5]^ Therefore, it is essential for anesthesiologists to choose appropriate agents to avoid prolonged extubation to improve the efficiency of OR. In Taiwan, Diagnosis Related Groups has taken a growing part in hospital billing system since 2010, and previous billing system is no longer valid for current anesthesia in the OR. Owing to predetermined payment for each case, the most cost-effective anesthetic technique should be determined. Anesthetic techniques with lower cost and shorter ACT are required to remain competitive in the operating field.^[1]^

Total intravenous anesthesia (TIVA) via a target-controlled infusion (TCI) system with propofol has been shown to provide more rapid emergence compared to other anesthetic techniques in several kinds of surgery.^[5–9]^ There were studies discussing the surgical condition in the field of functional endoscopic sinus surgery (FESS) comparing propofol via TCI and inhalation anesthetics.^[10,11]^ However, these studies did not compare the time required for extubation. FESS is one of the major surgeries performed by otolaryngologists in our hospital, so larger amount of data could be acquired. Therefore, we performed this retrospective study to compare the ACT between TIVA with propofol/fentanyl and DES anesthesia in FESS.

## 2 Material and methods

### 2.1 Patients

This study was approved by the Ethics Committee (TSGHIRB No: 100–05–168) of Tri-Service General Hospital, Taipei, Taiwan (Chairperson, Professor Pauling Chu) on August 29, 2011.

This retrospective study retrieved information from the electronic database and the medical records of Tri-Service General Hospital (TSGH; Taipei, Taiwan, Republic of China). A total of 717 patients (ASA class I∼III) who received elective FESS under TIVA or desflurane (DES) from January 2010 to December 2011 was included. For the purposes of this study, the following times (minutes) were calculated: waiting for anesthesia time, arrival in the OR to anesthesia was introduced; surgical time, incision to surgical completion and application of dressings; anesthesia time, start of anesthesia to extubation; extubation time, surgery complete and dressings applied to extubation; total OR stay time, arrival in the OR to departure from the OR; postanesthesia care unit (PACU) stay time, arrival in the PACU to discharge from the PACU to the general ward; and ACT, arrival in the OR to discharge from the OR. All time intervals were documented as electrical medical records by an OR nurse and were confirmed with the operator and the presiding anesthesiologist. Exclusion criteria were patient younger than 18 years, emergent surgeries, combined inhalation anesthesia with TIVA, other inhalation anesthesia besides DES, failure to extubate, patient not sent to the PACU, or incomplete data.

### 2.2 Patient groups

No medication was administered before induction of anesthesia; however, regular monitoring, such as electrocardiography (lead II) and measurement of pulse oximetry, noninvasive blood pressure, respiratory rate, and end-tidal carbon dioxide pressure (EtCO_2_), was performed. In all patients, anesthesia was induced with propofol and fentanyl. The patients were then intubated and maintained with the anesthetics DES or propofol and the analgesic fentanyl.

TIVA was induced using intravenous (i.v.) fentanyl (2 μg/kg) and 2% lidocaine (1.5 mg/kg). Subsequently, continuous infusion of propofol (Fresfol 1%) was delivered using Schneider kinetic model of TCI (Fresenius Orchestra Primea; Fresenius Kabi AG, Bad Homburg, Germany) with the effect-site concentration (Ce) of 4.0 μg/mL. For DES anesthesia, the patients were induced with i.v. fentanyl (2 μg/kg), 2% lidocaine (1.5 mg/kg), and propofol (1.5–2 mg/kg). When patients lost consciousness, 0.6 mg/kg of rocuronium was administered, followed by endotracheal intubation and administration of i.v. dexamethasone (5 mg) to prevent postoperative nausea and vomiting (in all patients). For TIVA, anesthesia was maintained using TCI with a propofol Ce of 3 to 4 μg/mL under an oxygen flow of 300 mL/min. In patients anesthetized with DES, anesthesia was maintained using 8% to 12% DES (inhaled concentration) in an oxygen flow of 300 mL/min under a closed system without nitrous oxide. Ce for TCI propofol was adjusted at the range of 0.2 μg/mL and DES 0.5% according to the hemodynamics. If 2 increments or decrements were unsuccessful, the range of Ce for TCI propofol and DES was increased to 0.5 μg/mL or 2%, respectively. Ventilation rate and maximum airway pressure were adjusted to maintain the EtCO_2_ pressure at 35 to 45 mmHg. Cisatracurium (2 mg) or rocuronium (10 mg) was administered as required to antagonize the return of neuromuscular function.^[5–9,12–17]^

At the end of the operation, DES or propofol was discontinued, and the lungs were ventilated with 100% oxygen at a fresh gas flow of 6 L/min. When the patient regained consciousness with spontaneous and smooth respiration, the endotracheal tube was removed.^[5–9,12–17]^ We defined an extubation time ≥15 minutes as prolonged extubation.^[3,18–20]^

### 2.3 Statistic analysis

Data are presented as the mean and standard deviation (SD), number of patients, or percentage. Demographic and perioperative data were compared using Student *t* test. Categorical data were compared using *χ*^2^ test. A *P* value of <.05 was considered statistically significant. Multiple linear regression analysis was performed to assess the association between variables influencing extubation time. Statistical analyses were done using SPSS software v.21.0. (IBM SPSS Statistics, IBM Corporation, Chicago, IL).

## 3 Result

**Figure 1:**
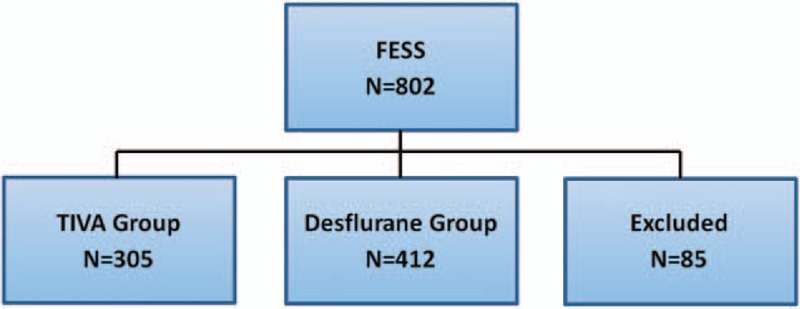
The flow diagram.

Eighty-five patients were excluded from the analysis: 56 patients received sevoflurane anesthesia, 18 patients received combined inhalation anesthesia with propofol, and 11 patients with incomplete data (Fig. 1).

Our study included 717 patients, of which 412 received DES and 305 received TIVA anesthesia. There were no significant differences in patient demographics. The extubation time was faster for TIVA group (8.8 [3.5] vs. 9.6 [4.0] minutes; *P* = .003) percentage of prolonged extubation was significantly higher in DES group (13.6% vs. 7.5%, risk difference 6.1%, *P* < .001). The time from the end of surgery to exit OR was also shorter in the TIVA group (15.7 [4.6] vs. 16.6 [5.0] minutes, *P* = .022). However, the surgery, anesthesia, ACT, total OR stay, and PACU times showed no differences between groups (Table 1).

**Table 1:**
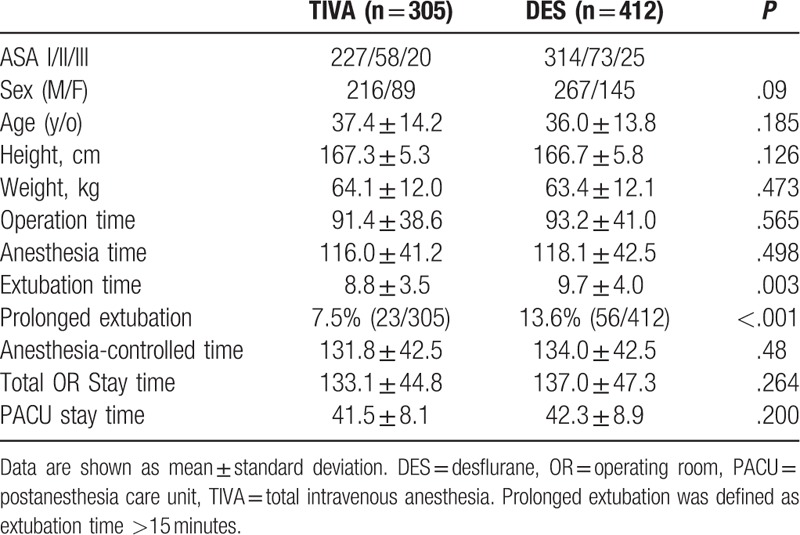
Patient's characteristics and operation room time measurment between TIVA and DES group.

The result of multiple linear regressions comparing extubation time between several variants was shown in Table 2. Total anesthesia time, surgical time, and groups are factors that contribute to extubation time. The result also showed that patients with shorter anesthesia and surgical time and TIVA have faster emergence. Age, sex, body weight, and height of the patients had no significant influences.

**Table 2:**
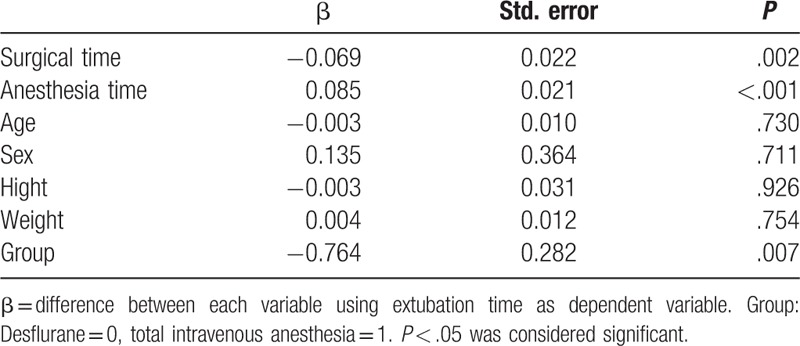
Comparisons of extubation time between several variants by multiple linear regressions.

## 4 Discussion

The major findings in this retrospective study show that propofol-based TIVA by TCI reduced the mean time to extubation and the risks of prolonged extubation relative to DES anesthesia in patients undergoing FESS. These findings were consistent with several of our previous studies showing that general anesthesia using TCI system with propofol could achieve faster extubation than using DES in different surgeries.^[5–9,12,14,15]^

In our study, the average extubation time was 0.8 minutes faster in TIVA group, and the average OR exit time was 0.9 minutes earlier in TIVA group. Although the differences were statistically significant, the time saved may not be sufficient for additional operation. McIntosh et al^[21]^ revealed that each 5-minute reduction in intraoperative time should be treated as reducing costs, and the reduction is approximately 20% larger than the cost per 5 minutes of OR time. However, the reduction of anesthesia related time in OR does not necessarily equal to improvement of OR productivity unless there are sufficient case-numbers to fill a workday. Therefore, the reduction in ACT, as reported in our study, may not be sufficient to schedule additional operation and could be reasonably treated as having no economic benefit.^[22]^

We also reported a significant lower percentage of prolonged extubation in TIVA group. Prolonged extubation would decrease OR efficiency and occurred in 15% of all cases.^[3,18]^ Therefore, monitoring the incidence of prolonged extubation was recommended as an economic measure.^[19]^ Because prolonged extubation after surgery would cause slowing of work flow, having OR members staying idly waiting for extubation, and the surgeon have to wait longer for the next operation.^[18,22]^ When surgeons rate anesthesiologists’ attributes on a scale from 0 = “no importance" to 4 = “a factor that would make me switch groups/ hospitals," their average score is 3.9 for “patient quick to awaken."^[23]^ Epstein et al^[20]^ studied the relationship between prolonged extubation and OR cost, and concluded that prolonged extubation time should be treated as resulting in proportionally increased variable costs. The risk factors of prolonged extubation including prone position, prolonged surgical time, significant blood loss, larger volume of crystalloid and colloid infusion, procedure, or surgeon. ^[20,24]^ In addition, more than half of the cases with prolonged extubation occurred during cases on regular workdays and in an OR with >8 hours of cases and turnover.^[23]^ The result was consisted with the previous reports, as we showed prolonged extubation was associated with anesthetic and surgical time. Another study conducted by the same group showed that the mean time from end of surgery to exit OR is at least 12.6 minutes longer in cases with prolonged extubation.^[19]^ In our study, we showed that the mean time to departure from OR to PACU was 15.7 and 16.6 minutes in TIVA and DES, respectively, which might be because of the paramedical factor.

The influences of different anesthetic agents and techniques on OR stay time have been extensively studied to provide better combination of anesthetic agents and anesthesia techniques to reduce OR cost without affect patient safety. Propofol has become popular in general anesthesia, especially in the ambulatory surgery or examine procedures. It is often used in combination with remifentanil because both drugs provide rapid induction and emergence with faster recovery of normal activity.^[25,26]^ However, remifentanil will become available in Taiwan recently, and propofol is cheaper in Taiwan than it is in America or Europe.^[27]^ Several previous studies have shown that general anesthesia under TIVA by TCI with propofol and fentanyl was cost-saving and achieved faster emergence in both short-term and long-term surgery when compared with DES and sevoflurane anesthesia.^[7,10]^

Some studies compared inhalation anesthesia with propofol-based TIVA and failed to show any significant clinical difference in terms of extubation.^[25,28,29]^ Dolk et al^[30]^ reported that there was shorter emergence time and reduced drug costs for DES anesthesia compared with propofol delivered by TCI in knee surgery. The differences may have been caused by using nitrous oxide as an adjuvant to anesthetics, which reduced the requirement of DES during maintenance period and facilitate early emergence. Another study reported by Mahli et al^[29]^ showed that there was no significance in extubation time between propofol-based TIVA by TCI system and DES anesthesia in ear, nose, and throat surgery. The difference might be because of the fact that DES maintenance flow rate of oxygen was different: 1∼4 L/min versus 300 mL/min in our study. For the purpose of smooth emergence, we turn off anesthetics later than in breast, gynecologic, ear, nose, and throat and spine surgeries to prevent coughing and straining during extubation; this may take several minutes. In addition, using close circuit anesthesia in the DES group would also prolong the neuromuscular blockade and delay the extubation times.^[31]^ The gas flow during emergence may also affect the time of extubation in DES group. As a retrospective study, the cases we collected were performed under routine clinical protocol, using 6 L/min of fresh gas flow during emergence. In our serial retrospective studies^[5–8,13–15]^ TIVA delivered by TCI system has the advantage in predicting the propofol effect-site concentration at return of consciousness.^[16,32–34]^ Moreover, TCI system could calculate the time required to reach such concentration, and therefore improve the OR efficiency. The dosage of opioids may also have an effect on extubation time. Fentanyl 2 to 6 μg/kg intravenous bolus injection is the suggested dose for anesthesia induction. FESS is considered as a less invasive procedure; therefore, we choose the lower limit of suggested dosage for our clinical practice. Finally, we have a much larger numbers of cases (a total of 717 vs. 40 cases), which may decrease investigators’ bias and reflect the better reality of our clinical practice.

## 5 Limitations

Our study has several limitations. First, retrospective study may lead to bias considering standardization and comparability of study groups. However, considering the purpose of this study, retrospective analysis of data offered a major advantage, namely that anesthetic management was performed by attending anesthesiologist according to clinical demands and was not by a study protocol. The study, performed under clinical conditions, reflects more precisely the clinical relevant benefit.

Second, hemodynamic profiles between these 2 groups were not recorded and compared in our study. Mahli et al^[29]^ reported that mean arterial blood pressure and heart rate were higher in TIVA group than in DES group. We do not have solid data, but this result differs from our clinical experience, although we use intravenous fentanyl bolus injection rather than remifentanil administrated with TCI system. In addition, in our clinical practice, the fentanyl dosage was higher in the TIVA group than the DES group.^[5,6,9,12,13,15]^

Finally, we did not use anesthesia depth monitors such as bispectral index (BIS) in our common practice.^[5,7,8,13–16]^ Because, in the practice of most anesthesiologists in Taiwan, BIS is not routine used during minor surgeries such as breast cancer surgery or FESS. Moreover, before our collected data period, many studies have suggested an absence of benefit of BIS monitoring during clinical anesthesia. However, in 2014, Punjasawadwong et al^[35]^ concluded that BIS reduced time for eye opening, response to verbal command, time to extubation, time to orientation, and reduced anesthetics requirement. Recently, Stein et al^[36]^ suggested that processed EEG monitors may be a useful adjunct undergoing a TIVA to prevent awareness but the cost was increased. Therefore, the cost effect of BIS still needs to be investigated in the low-risk patients (younger and health patients) who are receiving minor surgery (such as breast surgery and FESS) and short time anesthesia especially in TIVA.

## 6 Conclusion

Our results showed that propofol-based TIVA by TCI reduced the mean time on extubation and reduced the incidence of prolonged extubation relative to DES in FESS. Decreased prolonged extubation may have potential benefit on OR efficiency, whereas the significant statistic reduction of extubation time may not have a clinical effect on increasing OR efficiency in our retrospective study.
